# Early gut microbiome composition of very preterm infants randomised to receive human milk volumes of 60 ml/kg/day or more within the first 36 hours after birth

**DOI:** 10.1038/s41390-025-04456-5

**Published:** 2025-10-02

**Authors:** Ariel A. Salas, Christopher J. Stewart, Gregory R. Young

**Affiliations:** 1 Division of Neonatology, University of Alabama at Birmingham, Birmingham, AL USA; 2 Translational and Clinical Research Institute, Newcastle University, Newcastle, UK

## Abstract

**Background:**

Early and increased exposure to human milk combined with minimal exposure to parenteral nutrition could reduce hospitalisation costs, improve postnatal growth, and influence the diversity of the gut microbiome.

**Methods:**

Faecal samples from very preterm infants randomised to receive either 60 to 80 ml/kg/day (intervention group) or 20 to 30 ml/kg/day (control group) of human milk within the first 36 h after birth were collected at approximately postnatal day 14. After trial completion, samples were analysed by 16S rRNA gene sequencing to determine early differences in the gut microbiome between the randomisation groups using adjusted models.

**Results:**

We analysed faecal samples from 95 infants with a median gestational age of 31 weeks (mean birthweight: 1487 g). In adjusted analyses, taxonomic richness and Shannon alpha diversity were not significantly higher in the intervention group. No significant differences in microbial diversity composition between samples (i.e., beta diversity) were found. Four distinctive de novo community clusters were identified during the trial, but they did not differ according to randomisation groups.

**Conclusion:**

Early and increased exposure to human milk shortly after birth does not appear to increase the richness and diversity of the gut microbiome by postnatal day 14 in very preterm infants.

**Trial registration:**

ClinicalTrials.gov identifier: NCT04337710

**Impact:**

- In very preterm infants, early and increased exposure to human milk and its bioactive components did not alter gut microbiome richness or diversity by postnatal day 14.
- Randomisation strengthens microbiome analyses by limiting confounding in human milk feeding trials.

## Introduction

The early development of the gut microbiome could play a role in shaping health outcomes in preterm infants, who are particularly vulnerable to microbial dysbiosis due to their immaturity and frequent exposure to antibiotics, parenteral nutrition, and delayed enteral feeding.^1,2^ The early establishment of full enteral nutrition, which minimises exposure to parenteral nutrition, has been hypothesised to promote more favourable microbial colonisation by facilitating earlier and more consistent exposure to enteral substrates that support commensal microbial growth in the gut; however, clinical data to confirm this hypothesis remain limited and inconclusive.^3^

Despite emerging evidence that early enteral feeding is safe and associated with improved clinical outcomes,^4^ its impact on the early gut microbiome composition and diversity in very preterm infants remains unclear.^5–8^ Given the potential implications of early microbiome development for gastrointestinal, metabolic, and immunological health,^9^ understanding how the early provision of more human milk volumes and the subsequent reduction in the time to establish full enteral nutrition affects microbial colonisation patterns is of increasing importance.

In this study, we analysed faecal samples from very preterm infants who were randomised to receive human milk volumes ≥60 ml/kg/day within the first 36 h after birth. We aimed to evaluate whether these higher feeding volumes influence the richness, diversity, and community structure of the gut microbiome by postnatal day 14.

## Methods

### Study design and participants

This analysis was conducted as part of a randomised clinical trial that compared early (intervention group) versus delayed (control group) feeding strategies in very preterm infants (ClinicalTrials.gov identifier: NCT04337710). Details of the trial are published elsewhere.^10^ Briefly, the trial was approved by the University of Alabama at Birmingham Institutional Review Board and written informed consent was obtained prior to randomisation. Eligible participants were infants born at 28–32 weeks of gestation who were clinically stable to receive enteral feeds shortly after birth. The primary outcome of the trial was the duration of full enteral feeding. Secondary outcomes included growth and body composition at postnatal day 14. A total of 102 infants were randomised. All study participants received either maternal or donor milk diets for the first 14 days after birth. Bovine-derived human milk fortifiers were added after the establishment of full enteral feeding. Routine evaluation of gastric residual volumes was not performed during the trial. While probiotics were routinely administered in other preterm populations during the trial, typically using preparations containing *Bacillus coagulans*, *Lactobacillus acidophilus*, *Bifidobacterium bifidum*, and *Bifidobacterium infantis*, they were not prescribed to all trial participants. Infants in the intervention group had significantly more full enteral feeding days within the first 28 days, greater fat-free mass accretion by postnatal day 14, improved length-for-age at discharge, and lower hospitalisation costs.^10^

### Sample collection and microbiome analysis

Faecal samples were collected from enroled infants at approximately postnatal day 14 (median: 13 days; IQR: 12–14). Samples were stored at −80 °C until processing. DNA was extracted using the Zymo D6010 Faecal DNA isolation kit (also referred to as Quick-DNA Fecal/Soil Microbe MiniPrep kit) from Zymo Research following the manufacturer’s instructions,^11,12^ and 16S rRNA gene sequencing generated by PCR amplification of the V4 region with uniquely barcoded primers was performed to characterise microbial communities.^3^ Sequences were processed using a validated bioinformatics pipeline to generate taxonomic profiles and diversity metrics.^11^ Filtering, denoising, and clustering of reads into Operational Taxonomic Units (OTUs) was done using DADA2. Taxon assignment was performed with Mothur ^13^ and the SILVA 16S rDNA gene database v132.^14^

Greater than 6500 reads were observed in all samples, at which point rarefaction curves were completely asymptotic, suggesting sufficient sequencing depth and comparable data between intervention groups (Supplementary Fig. S1). Read counts were converted to proportional abundances for analyses.

### Statistical analysis

Statistical analyses were performed in R.^15^ Alpha (rarefied taxonomic richness (*n* = 6500 reads) and Shannon diversity indices) and beta (Bray-Curtis dissimilarity) diversity metrics were calculated with the vegan package for community ecology (doi:10.32614/CRAN.package.vegan), and used to examine overall differences in microbiota composition between intervention groups. Generalised linear adjusted models were built for both Richness (negative-binomial) and Shannon diversity, including sex, race, delivery mode, siblings, maternal antibiotic exposure, steroid use, probiotic use, postnatal antibiotic exposure, and excessive fat mass (> −1 fat mass *z* score at postnatal day 14) as categorical covariates and gestational age, postmenstrual age, enteral feed volume at 14 days and maternal-to-donor milk ratio as continuous covariates. Birth weight (VIF = 27.09; R^2^ = 0.97; *P* = < 0.001) and birthweight Z-score (15.78; R^2^ = 0.94; *P* = < 0.001) were not included in models due to significant co-linearity with gestational age. The impact of clinical covariates on overall sample composition was assessed using PERMANOVA.

Clustering analyses were performed and gap statistic utilised to identify distinct de novo microbial community structures as previously described.^16^ Cluster membership was compared between feeding groups and other categorical variables using the chi-squared test. The association of taxonomic richness, diversity and other continuous covariates with cluster membership was compared using Kruskal-Wallis rank-sum test. Results were adjusted for multiple comparisons where appropriate using the Bonferroni method.

## Results

### Overview of the analytical cohort

Of the 102 infants enroled in the parent trial, 98 (96%) provided at least one faecal sample. After excluding three samples due to identification of two chromosomal anomalies and one congenital viral infection, 95 unique samples collected around postnatal day 14 were included in the microbiome analysis. The median gestational age of the cohort was 31 weeks (interquartile range [IQR]: 29–32), and the mean birth weight was 1487 g (standard deviation [SD]: ±330).

Baseline characteristics of the intervention (*n* = 47) and control (*n* = 48) groups were generally well balanced. While gestational age (*P* = 0.02), birth weight (*P* = 0.003), and birth weight z-score (*P* = 0.04) differed slightly between groups, sex distribution, mode of delivery, and exposure to antenatal steroids or early antibiotics were not significantly different (*P* > 0.05) (Table 1). The cumulative intake of human milk during the two weeks after birth was higher in the intervention group (Supplementary Fig. S2). Library size (median = 33,504; IQR = 24,084–41,539) did not differ significantly between groups (*P* = 0.23).

**Table 1:** Participant characteristics and sample availability

	Intervention group (*n* = 47)	Control group (*n* = 48)	*p*
Gestational age in weeks	31 (30–32)	30 (29–32)	**0.02**
Birthweight in grams	1580 (1370–1790)	1348 (1118–1590)	**0.003**
Z-score	−0.09 (−0.62–0.49)	−0.26 (−0.83–0.10)	**0.04**
Male sex, *n* (%)	21 (45)	24 (50)	0.69
White race, *n* (%)	24 (51)	27 (56)	0.81
Hispanic/Latino ethnicity, *n* (%)	9 (19)	9 (19)	1.00
Vaginal delivery, *n* (%)	22 (47)	18 (37)	0.41
Multiple gestation, *n* (%)	17 (36)	12 (25)	0.27
Maternal antibiotics, *n* (%)	26 (55)	24 (50)	0.68
No exposure to antenatal steroids, *n* (%)	5 (10)	4 (8)	0.66
Excessive fat mass, *n* (%)	24/46 (52)	15/42 (36)	0.14
Cumulative enteral intake volume up to postnatal day 14 (ml/kg)	1743 (1626–1841)	1618 (1477–1683)	**0.0004**
Cumulative maternal to donor milk ratio up to postnatal day 14 (%)	55 (5−77)	35 (3−88)	0.56
Exposure to postnatal antibiotics within the first 48 h after birth, *n* (%)	40 (85)	41 (85)	0.97
Exposure to antibiotics between postnatal days 3 and 14	11 (23)	2 (4)	0.006
Enteral supplementation with probiotics within the first week after birth, *n* (%)	17 (36)	33 (69)	0.002
Postnatal age at the time of stool sample collection in days	13 (12−13)	13 (12−14)	0.32
Postmenstrual age at the time of stool sample collection in weeks	33 (32−34)	33 (32−34)	0.07
Library size	35,920 (26,526−42,727)	31,094 (23,627−39,046)	0.23

Statistically significant *p* < 0.05 values are in bold.

Taxonomic analysis revealed that the gut microbiota was dominated by facultative anaerobes, consistent with typical colonisation patterns in preterm infants. The most abundant genera across all samples included *Enterobacteriaceae*, *Staphylococcus*, *Escherichia-Shigella*, and *Bifidobacterium*. A summary of microbial taxa with relative abundance >1% at postnatal day 14 is shown in Supplementary Fig. S3.

### Early enteral feeding has no impact on gut bacterial alpha diversity

**Fig. 1:**
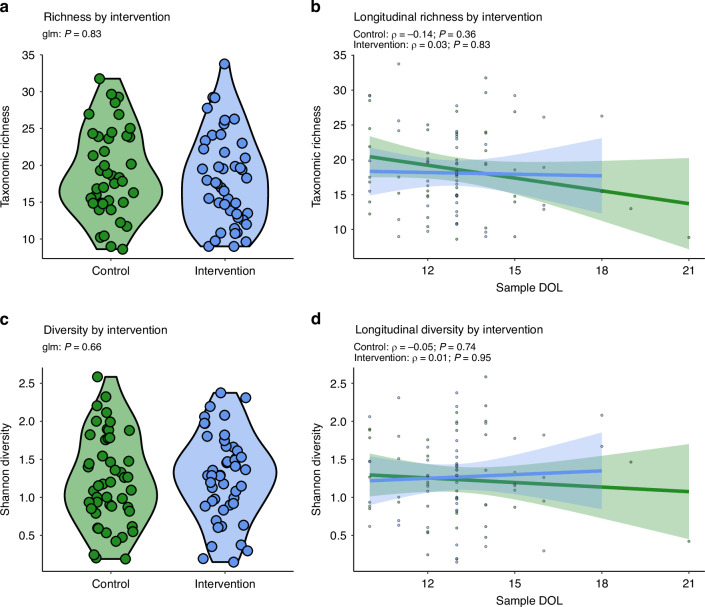
Alpha diversity. No significant difference in taxonomic richness **a**, **b** or Shannon diversity **c**, **d** was observed between enteral feed intervention groups. Violin plots **a**, **c** compare global alpha diversity values across all samples. Each point represents an individual sample with violin width linked to density of samples at each metric value. Scatter plots illustrate opposing trends in richness **b** and Shannon diversity **d** between intervention groups, though these associations were insignificant.

Alpha diversity was assessed using two complementary measures: rarefied taxonomic richness (number of unique OTUs) and the Shannon diversity index (which accounts for both richness and evenness of the microbial community). While richness was significantly greater in stools collected on earlier postnatal days, this effect diminished after excluding outliers (*n* = 2, Supplementary Fig. S4). Taxonomic richness was not significantly different between early (mean = 2.80; SE = 0.11) and late (mean 2.82, SE = 0.11) enteral feeding groups (adjusted mean difference: 0.7; *P* = 0.83) after adjusting for covariates (Fig. 1A, B). Shannon diversity was not significantly different between early (mean = 1.21, SE = 0.23) and late (mean = 1.21, SE = 0.24) enteral feeding groups in adjusted analyses (*P* = 0.61; Fig. 1C). Aside from the main trial, delivery mode was the only covariate significantly associated with either alpha diversity measure. Significantly greater Shannon diversity was observed in vaginally (mean = 1.03, SE = 0.23) than caesarean (mean = 1.33, SE = 0.25) delivered infants after adjusting for other covariates (*P* = 0.03; Supplementary Fig. S5).

### Early enteral feeding has no impact on overall gut bacterial composition

**Fig. 2:**
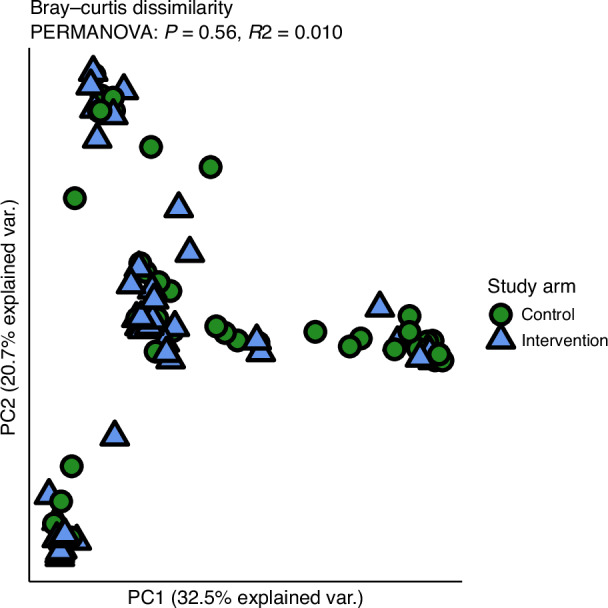
Beta-diversity: Bray-Curtis dissimilarity. No significant dissimilarity in overall community composition was observed between early and late enteral feeding intervention arms. Each point on the principal components plot represents an individual sample and is coloured and shaped by study arm.

To evaluate overall differences in microbial community structure, beta diversity was calculated using Bray-Curtis dissimilarity and visualised through principal coordinates analysis (PCoA). No distinct clustering was observed by early or late feeding groups, and PERMANOVA testing confirmed the absence of significant differences in community composition by intervention (*P* = 0.91; Fig. 2). These findings suggest that initiation of enteral nutrition with human milk volumes ≥60 ml/kg/day had no measurable impact on the overall structure of the gut microbiome at postnatal day 14, indeed, none of the covariates recorded in this study had any significant impact on the gut microbiome at the time of sampling (Supplementary Table S1).

**Fig. 3:**
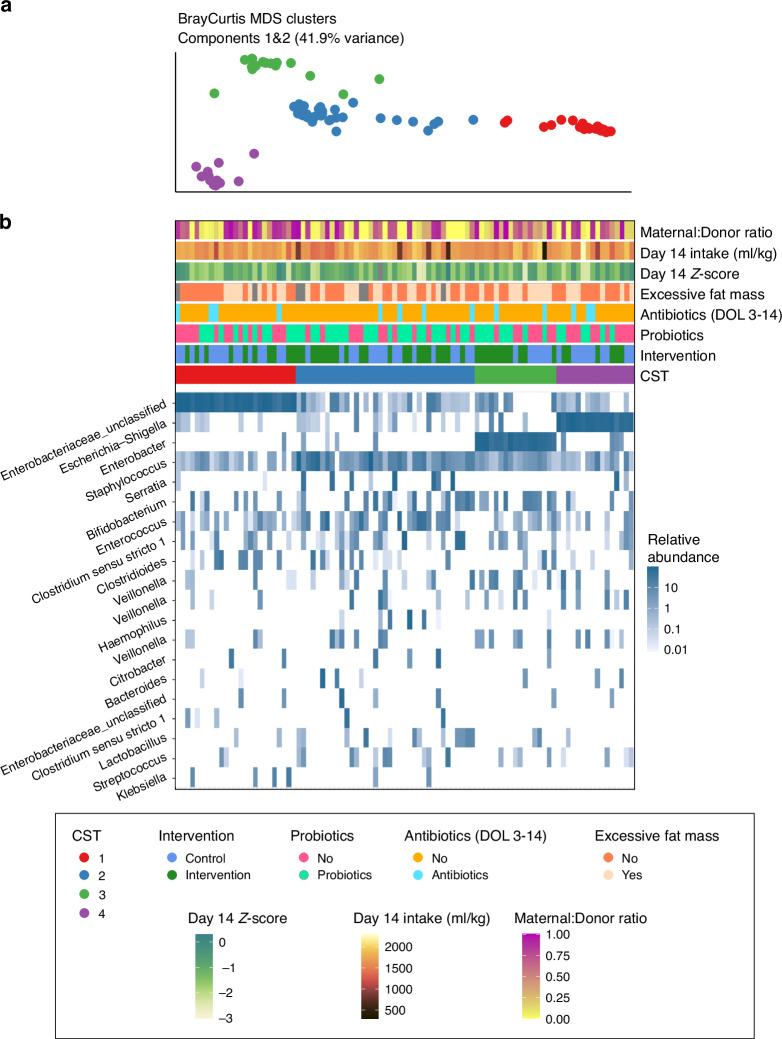
Bray Curtis MDS clusters. De novo clustering of individual sample microbial Bray-Curtis dissimilarity scores identified four community state types (CST) characterised by distinct microbial community compositions. Mutli-dimensional scaling **a** plots the overall composition of each sample as an individual point, coloured by CST grouping. Heatmap **b** shows distinct bacterial compositions between CSTs. The colour intensity of each tile represents the relative abundance of a single genus (rows) across samples (columns) (white = low, blue = high). Samples are arranged on the x-axis according to compositional similarity. CST grouping of each sample (column) is plotted as per colour scheme above the heatmap along with other clinical covariates. No CSTs were associated with intervention group or other clinical covariates. (Community state types: CST1 red, Enterobacteriaceae dominance, CST2 = blue, Staphylococcus and Enterococcus dominance, CST3 green, Enterobacter dominance, CST4 purple, Escherichia dominance).

Four distinct community state types (CSTs) were identified via unsupervised clustering of weighted Bray-Curtis dissimilarity scores (Fig. 3). Gap statistic values suggested the four CSTs, represented distinct compositional patterns across the cohort: CST 1 –Dominated by *Enterobacteriaceae;* CST 2 –A more even composition with *Staphylococcus* and *Enterococcus;* CST 3 –Enriched in *Enterobacter*; CST 4 –Enriched in Escherichia. CST 2 appeared to represent a transitional state between the other community types. The distribution of CSTs did not significantly differ by randomisation group (*P* = 0.25), indicating that the timing of full enteral nutrition did not drive the clustering of microbial community patterns. Exposure to either antibiotics or probiotics postnatally had no significant effect on richness, diversity, or CST membership. Adjusting analyses for postmenstrual age (instead of postnatal age in days or gestational age at birth) did not alter the findings.

## Discussion

In this randomised trial of very preterm infants, initiation of enteral nutrition with human milk volumes ≥60 ml/kg/day within the first 36 h after birth did not result in significant differences in gut microbiome richness, diversity, or overall community structure at postnatal day 14. These findings suggest that advancing to full enteral feeding three days earlier, while clinically safe and nutritionally beneficial, may not be sufficient on its own to alter early gut microbial development during the first two weeks after birth.

0Our results add to a growing body of literature indicating that early nutritional exposures are just one of many other factors that could potentially influence the development of the gut microbiome in preterm infants.^17^ Gammaproteobacteria dominance is a common feature of the early gut microbiome in preterm infants fed either maternal or donor milk,^7^ especially during the first weeks in the neonatal intensive care unit.^16,18–20^

Factors such as antibiotic exposure, probiotic use, mode of delivery, and the neonatal unit environment likely exert stronger or more proximal effects during the early postnatal period.^1,21^ In our cohort, the lack of significant differences in microbial diversity metrics between feeding groups persisted even after adjusting for these known covariates.

The four microbial community types identified in our cohort mirror those previously described in preterm populations, including patterns dominated by *Enterobacteriaceae* or *Staphylococcaceae*, both of which are commonly associated with antibiotic exposure and delayed colonisation by commensal anaerobes such as *Bifidobacterium*.^22^ The lack of difference in community type distribution between groups further supports the idea that feeding strategy alone, particularly in the first week of life, may have limited impact on early colonisation patterns under specific conditions. First, all infants received human milk–based diets, which are known to play a major role in shaping the gut microbiome regardless of the type of fortifier used.^23^ Second, the trial did not involve routine evaluations of gastric residual volumes; avoiding gastric aspiration has been associated with a more commensal-dominated microbiome.^24^ Third, no additional supplements, such as probiotics, were routinely used in study participants, though prior studies suggest that combining early nutrition with probiotics can accelerate microbial maturation.^25^ Fourth, feeding intolerance was uncommon, a clinical problem that often results in unfavourable microbial shifts.^26^ Finally, there were relatively few episodes of sepsis requiring systemic antibiotics during the trial, which may have reduced potential microbiome disruptions.^27^

Notably, our findings do not rule out potential longer-term effects of early enteral nutrition on the microbiome.^28^ The observed convergence in diversity and composition by postnatal day 14 may obscure transient differences that occur earlier, or longer-lasting differences that only emerge with extended feeding exposure. Future analyses including serial sampling across the first month of life^22^ and beyond may be better suited to detect these dynamics.

Our study has several strengths, including a randomised design, high sample collection rate, and adjustment for multiple potential confounders. However, it also has limitations. The sample size was relatively small. The timing of faecal sample collection at a single postnatal time point may have missed transient changes in microbial communities that could become more evident at later postnatal ages. Specifically, opposing temporal trends in richness and diversity of neonates exposed to early and late enteral feeding observed here suggest longitudinal tracking of the microbiome within these individuals may uncover intra-participant changes that this cross-sectional study was unable to identify with regression models adjusted for covariates that were inherently limited in resolution. Previous studies have shown initial reductions in taxonomic richness in the immediate postnatal period, preceding subsequent increases as the microbiome develops.^21^ Additionally, our analysis focused on taxonomic composition derived from amplification of the V4 region of the 16S rRNA gene, which does not capture functional microbial activity or strain-level differences that may also be important.

In conclusion, enteral nutrition initiated by postnatal day 2 with human milk volumes of 60 ml/kg/day or greater does not appear to significantly increase gut microbiome richness or diversity by postnatal day 14 in very preterm infants. These results highlight the highly dynamic and variable nature of the early preterm microbiome, suggesting that single interventions during this period may have limited impact. Given the clinical stability and feeding tolerance observed, it may be more informative to focus on longitudinal microbiome changes and their associations with long-term health, rather than early snapshots that may not fully capture relevant microbial trajectories or outcomes.

## Supplementary information

Supplementary information

## Data Availability

The datasets generated during and/or analysed during the current study are available from the corresponding author on reasonable request.
