# Supplementary material for: Early gut microbiome composition of very preterm infants randomised to receive human milk volumes of 60 ml/kg/day or more within the first 36 hours after birth
Source: Pediatr Res. 2025 Oct 2;99(5):1964–9. doi: 10.1038/s41390-025-04456-5 (PMC13221289; doi:10.1038/s41390-025-04456-5)
Supplement: Supplementary file 1 — Supplementary information [file 41390_2025_4456_MOESM1_ESM.pdf]

Supplemental Figure S1. Library size and rarefaction curves according to randomization groups.

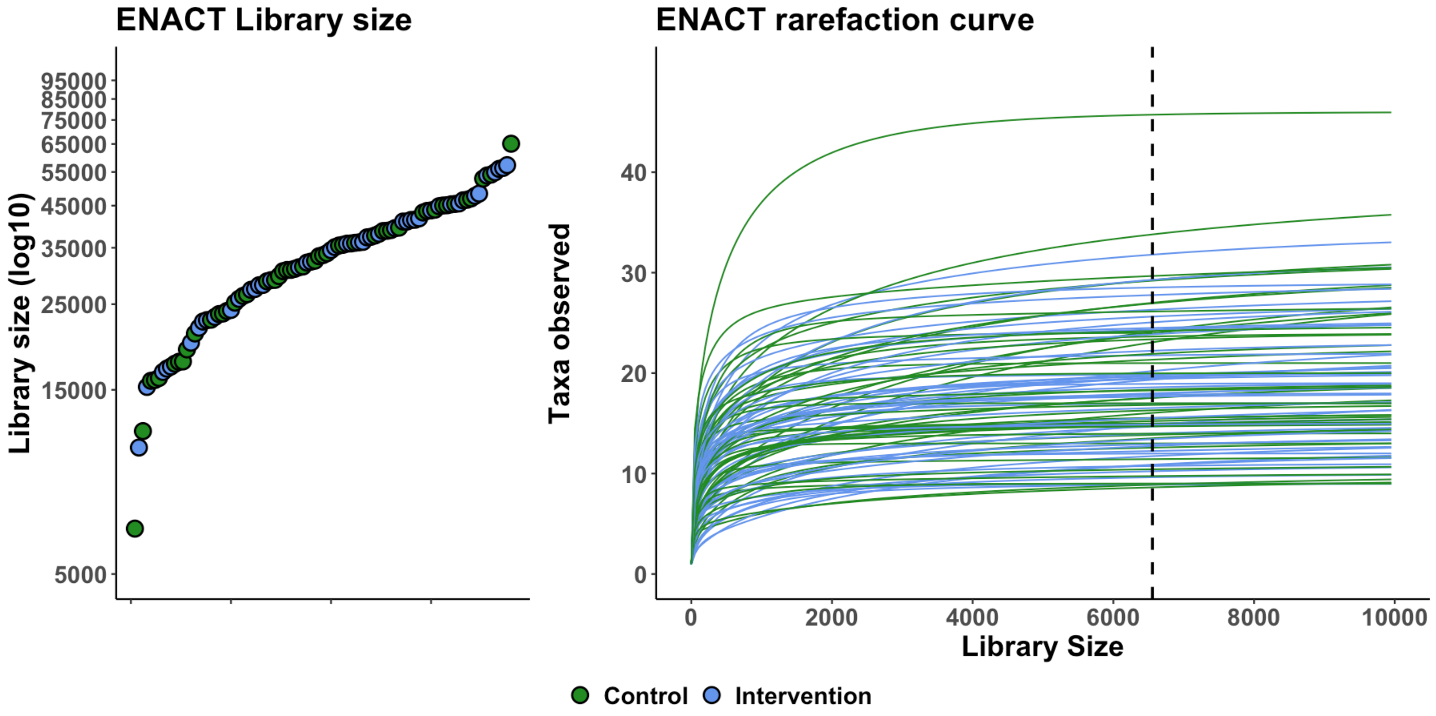

No significant difference in bacterial 16S rRNA gene library size was observed between stool samples from participants randomized into each intervention arm ($P = 0.23$). Each point of the scatter plot (A) or line of the rarefaction curve (B) represents an individual sample, coloured by intervention group. A minimum of 6555 reads were observed across all samples. Rarefaction depth (dashed line in rarefaction curve) was set at 6500 reads, by which point all curves have achieved asymptote, indicating sufficient sampling depth of each sample for analysis.

Supplemental Figure S2. Cumulative enteral intake of human milk in first two postnatal weeks

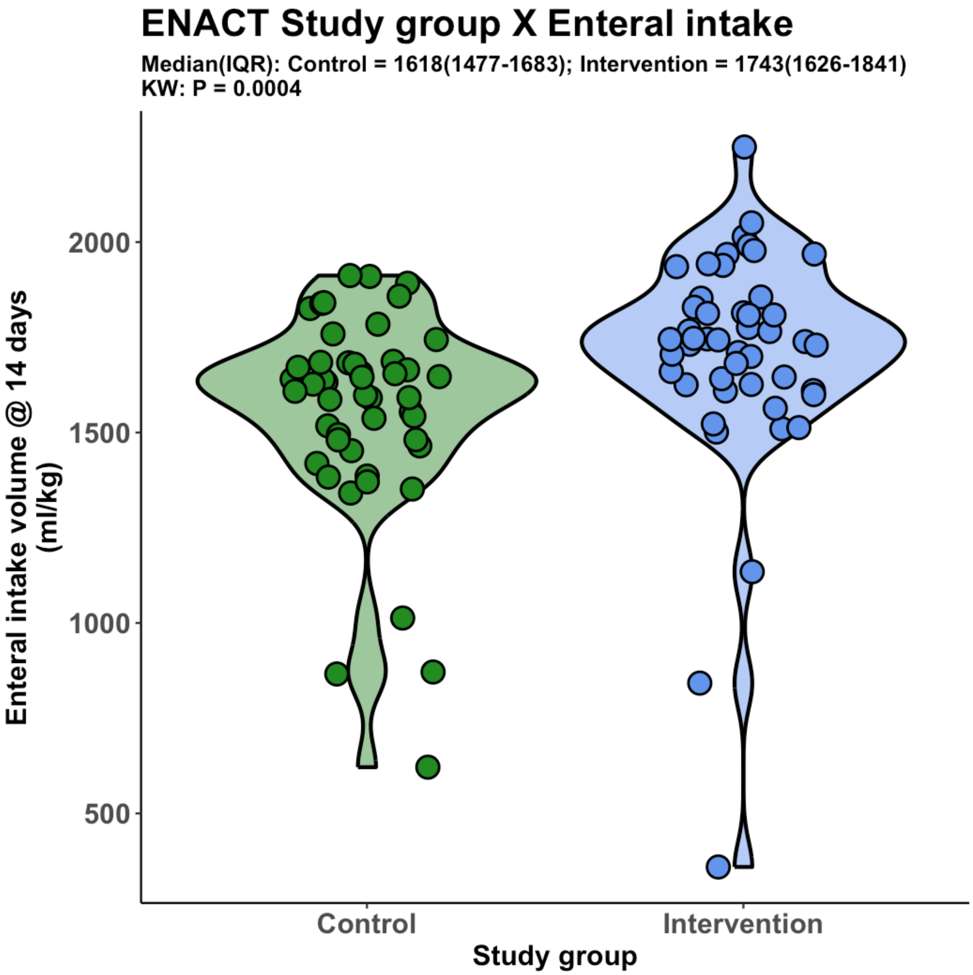

A significant difference in enteral intake of human milk within the first two weeks of life was observed between control (green) and intervention (blue) trial arms (P = 0.0004). Each point represents an individual participant. Violin plot widths are linked to density of participants receiving milk volumes.

Supplemental Figure S3. Gut microbiome composition in very preterm infants at postnatal day 14.

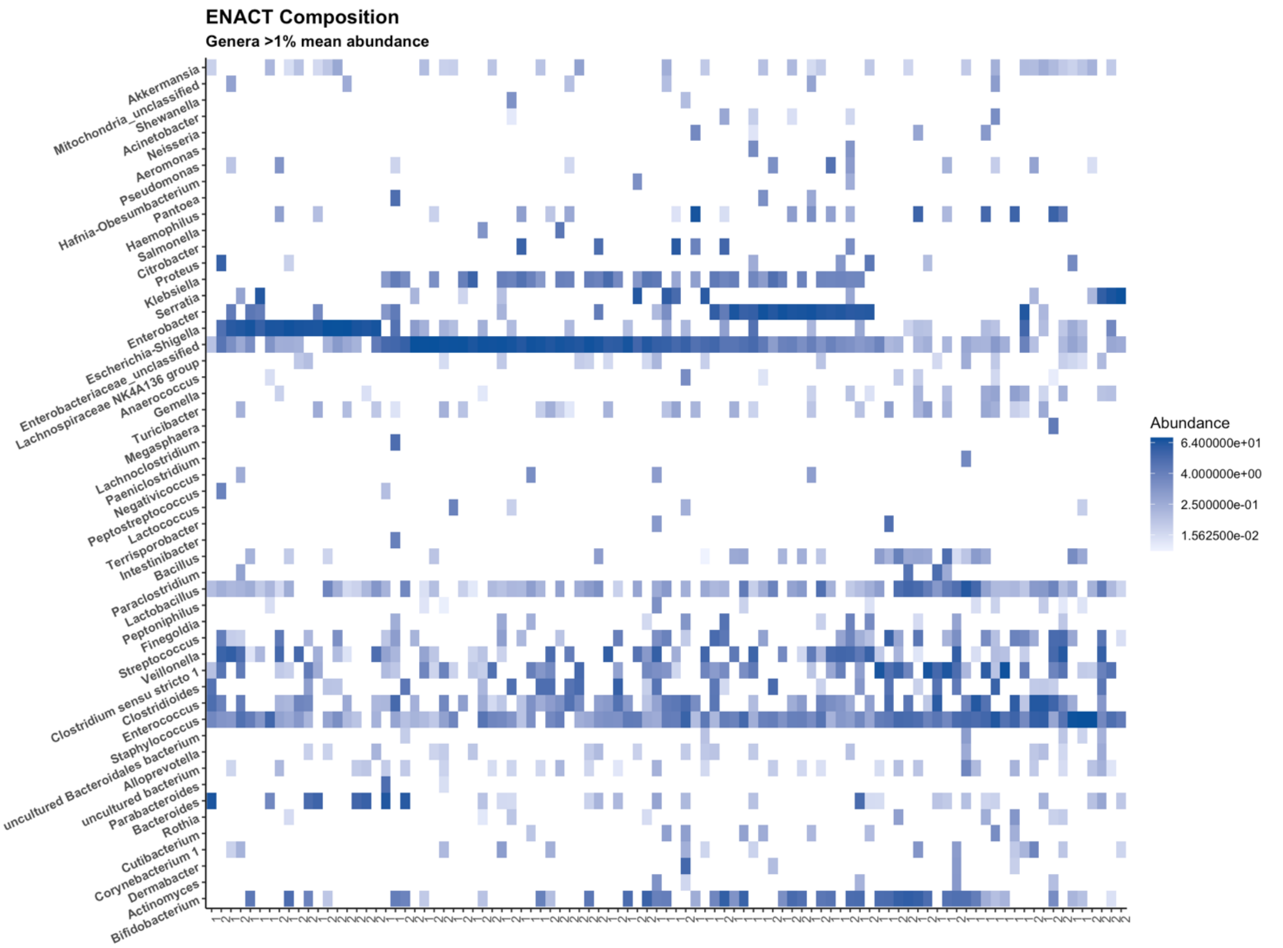

Taxonomic composition of the most abundant genera (>1% mean abundance) observed in all samples. Tile colour intensity indicates proportional abundance of each genus. Samples are arranged on x axis by compositional similarity (Bray-Curtis) and taxa arranged on y axis by Phylum. Samples are labelled on the x axis by intervention group (1 = control; 2 = intervention)

Supplemental Figure S4. Association of taxonomic richness with postnatal age at the time of stool sample collection

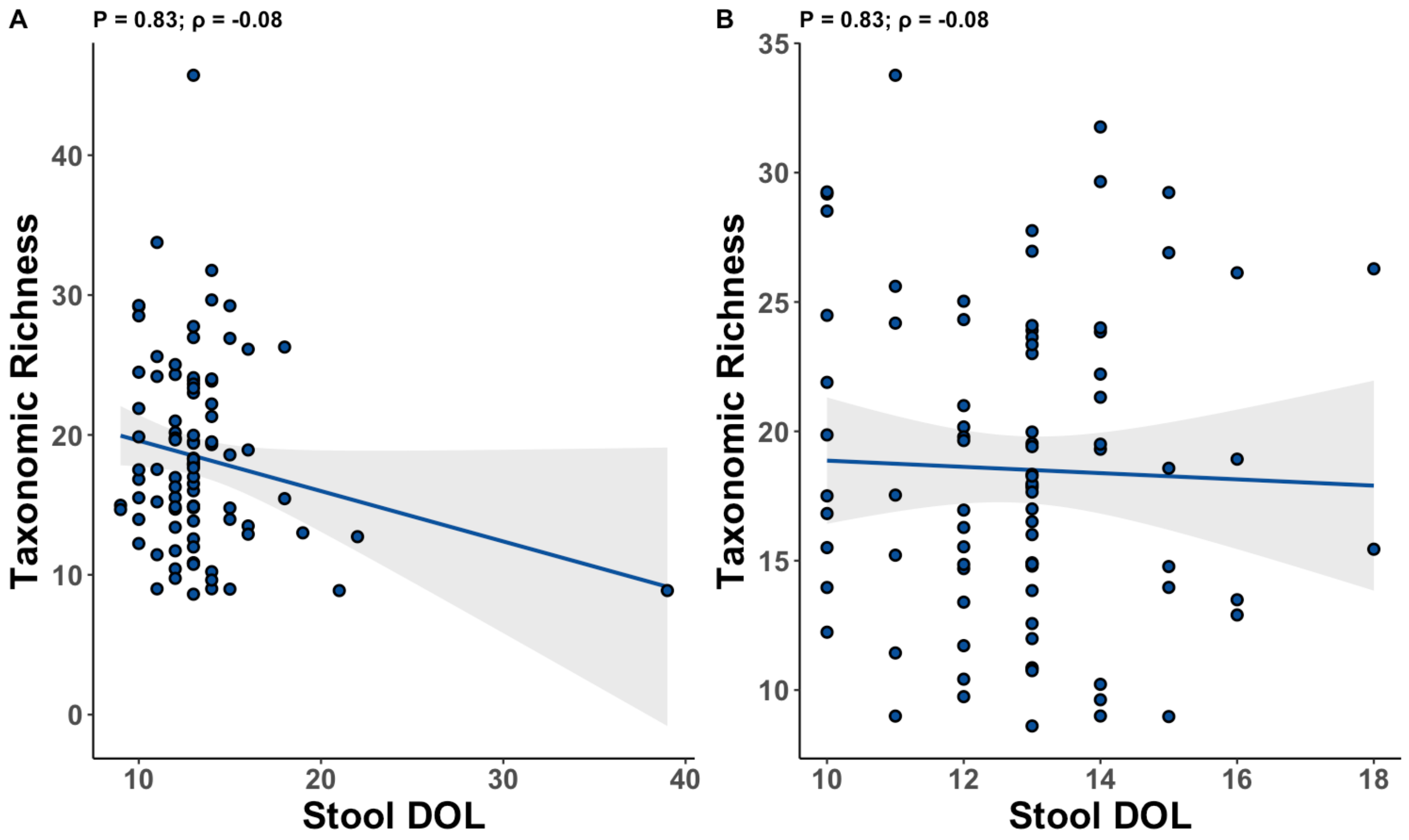

Taxonomic richness is significantly negatively associated with stool sample collection postnatal day of life in total cohort (n = 95, A) but not after removal of outliers (n = 93, B). Outliers were removed where observed taxonomic richness was > 40 or post-natal day of stool collection >30. Each point represents an individual

Supplemental Figure S5. Shannon diversity differences by delivery mode

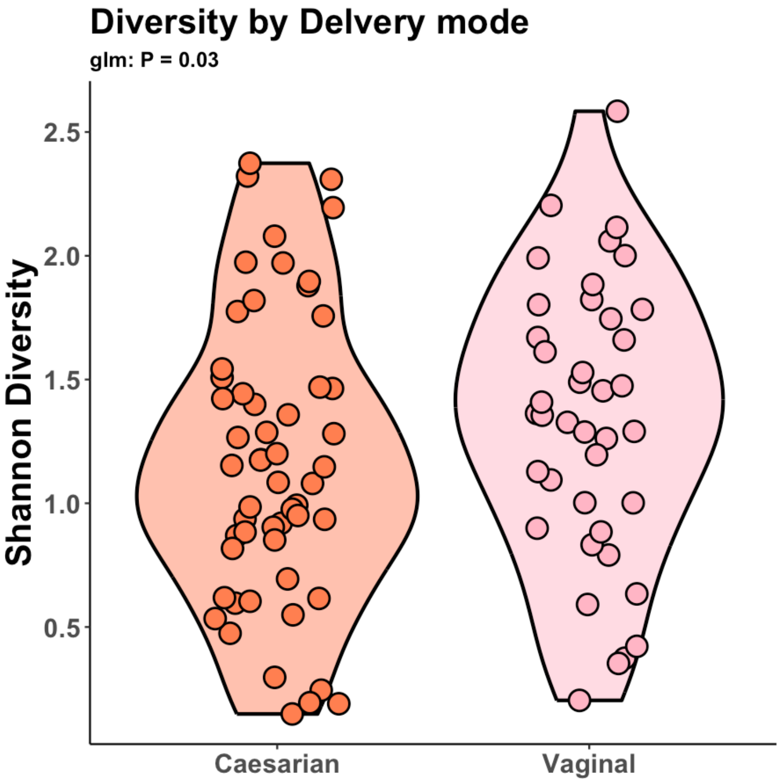

After controlling for other covariates, delivery mode was the only covariate that was significantly associated with Shannon diversity. Lower diversity was observed in stools of participants delivered via caesarean section (median = 1.15; IQR = 0.84 – 1.49) than those delivered vaginally (median = 1.36; IQR = 0.976 – 1.75)(*P* = 0.03).

Supplemental Table S1. Permutational Analysis of Variance

	R2	F	Pr(>F)
Stool DOL	0.01958	1.6614	0.074
Study group	0.0059	0.5006	0.894
Sex	0.014	1.188	0.243
Race	0.01354	0.5744	0.931
Gestational age	0.02477	0.7007	0.866
Delivery Mode	0.01468	1.2461	0.225
Multiple gestation	0.01413	1.1991	0.288
Maternal Abx	0.00967	0.8209	0.568
Steroids	0.02604	1.1051	0.322
Excessive fat mass	0.01038	0.8812	0.491
14d enteral intake	0.01656	1.4053	0.185
Maternal ratio	0.00957	0.8123	0.606
Residual	0.81307		

F = F statistic; P = probability; DOL = Day of life; 14d enteral intake = Enteral intake volume at 14 days of life
